# Candesartan Attenuates Cisplatin-Induced Lung Injury by Modulating Oxidative Stress, Inflammation, and TLR-4/NF-κB, JAK1/STAT3, and Nrf2/HO-1 Signaling

**DOI:** 10.3390/ph15101222

**Published:** 2022-10-02

**Authors:** Ahmed M. Atwa, Omnia A. M. Abd El-Ghafar, Emad H. M. Hassanein, Somya E. Mahdi, Ghadir A. Sayed, Reem S. Alruhaimi, Haifa A. Alqhtani, Mohammed F. Alotaibi, Ayman M. Mahmoud

**Affiliations:** 1 Department of Pharmacology and Toxicology, Faculty of Pharmacy, Egyptian Russian University, Cairo 11829, Egypt; 2 Department of Pharmacology and Toxicology, Faculty of Pharmacy, Nahda University, Beni-Suef 62521, Egypt; 3 Department of Pharmacology and Toxicology, Faculty of Pharmacy, Al-Azhar University, Assiut 71524, Egypt; 4 Department of Physiology, Faculty of Medicine, Zagazig University, Zagazig 44519, Egypt; 5 Department of Biochemistry, Faculty of Pharmacy, Egyptian Russian University, Cairo 11829, Egypt; 6 Department of Biology, College of Science, Princess Nourah bint Abdulrahman University, Riyadh 11671, Saudi Arabia,; 7 Physiology Department, College of Medicine, King Saud University, Riyadh 11461, Saudi Arabia; 8 Physiology Division, Zoology Department, Faculty of Science, Beni-Suef University, Beni-Suef 62514, Egypt; 9 Department of Life Sciences, Faculty of Science and Engineering, Manchester Metropolitan University, Manchester M1 5GD, UK

**Keywords:** chemotherapy, candesartan, lung injury, oxidative stress, inflammation

## Abstract

Cisplatin (CIS) is an effective chemotherapeutic agent against different cancers. The use of CIS is associated with acute lung injury (ALI) and other adverse effects, and oxidative stress and inflammation were implicated in its toxic effects. Candesartan (CAN), an angiotensin II (Ang II) receptor blocker, showed beneficial effects against oxidative stress and inflammation. Therefore, this study investigated the potential of CAN to prevent CIS-induced oxidative stress, inflammation, and lung injury in rats, pointing to the involvement of TLR4/NF-κB, JAK1/STAT3, PPARγ, and Nrf2/HO-1 signaling. The rats received CAN (5 mg/kg) for 10 days and were challenged with a single dose of CIS (7 mg/kg) on day 7. CIS caused injury to the alveoli and the bronchial tree, increased lipid peroxidation, nitric oxide, myeloperoxidase, TLR-4, NF-κB p65, iNOS, TNF-α, IL-6, IL-1β, and caspase-3, and decreased cellular antioxidants and IL-6 in the lungs of rats. CAN effectively prevented tissue injury, suppressed TLR-4/ NF-κB signaling, and ameliorated oxidative stress, inflammatory markers, and caspase-3 in CIS-administered rats. CAN enhanced antioxidants and IL-10, decreased Ang II, increased Ang (1–7), suppressed the phosphorylation of JAK1 and STAT3, and upregulated SOCS3 in CIS-administered rats. These effects were associated with the downregulation of Keap1 and enhanced Nrf2, GCLC, HO-1, and PPARγ. In conclusion, CAN prevented CIS-induced lung injury by attenuating oxidative stress, suppressing TLR-4/NF-κB and JAK1/STAT3 signaling, Ang II, and pro-inflammatory mediators, and upregulating PPARγ, and Nrf2/HO-1 signaling.

## 1. Introduction

Platinoids are platinum-based chemotherapeutic drugs commonly used against many malignancies in up to 50% of cancer patients. Cisplatin (CIS) is one of the most common platinoids used to treat various cancers, including lung, cervical, breast, gastric, and others [1,2]. CIS increases the generation of free radicals and interferes with DNA replication through cross-linking, leading to apoptotic cell death [1]. The use of CIS is associated with several adverse effects, including acute lung injury(ALI) which can be life-threatening [3,4], thereby limiting its clinical applications. Injury to the alveolar-capillary membrane and non-cardiogenic pulmonary edema are the characteristic clinical features that can culminate in hypoxemia and mortality in ALI [5]. The mechanism underlying CIS-associated ALI is still not fully understood; however, oxidative stress and inflammation have been implicated in its toxic effect on the lung as well as other organs [6,7,8,9]. Eosinophilic pneumonia is one of the adverse effects of CIS reported in a case report [10]. CIS provoked inflammatory cell infiltration and thickening of the interalveolar septa, reactive oxygen species (ROS) generation, lipid peroxidation (LPO), apoptosis, and ciliary fragmentation in the lung of mice [11].

Oxidative injury mediates multiple inflammatory mechanisms, leading to tissue damage. ROS can activate nuclear factor-kappaB (NF-κB) that controls the release of cytokines and mediates cell damage [12]. ROS are also involved in Toll-like receptor (TLR)-4-associated activation of NF-κB [13] and the release of cytokines, including tumor necrosis factor (TNF)-α, interleukin (IL)-1β, and IL-6 [14]. TLR-4 has been linked to ALI through its implication in promoting inflammation in response to infectious and non-microbial stimuli [15,16]. The produced pro-inflammatory mediators work in concert with ROS to provoke cell death and can also activate multiple signaling pathways resulting in tissue injury. Upon binding to their receptors, cytokines activate and dimerize the receptors resulting in tyrosine phosphorylation of Janus tyrosine kinases (JAKs) and the subsequent activation of signal transducer and activator of transcriptions (STATs) via tyrosine phosphorylation. STATs dimerize and translocate to the nucleus to activate the transcription of target genes involved in inflammatory response, differentiation, proliferation, and apoptosis [17,18]. Oxidative stress and IL-6 have been shown to activate STAT3 through a JAK-dependent mechanism [19], findings that were supported in our previous studies [20,21,22]. Therefore, enhancement of the antioxidant defenses and attenuation of ROS and inflammation could be effective against CIS-induced lung injury.

The activation of the nuclear factor erythroid 2-related factor 2 (Nrf2) is effective in attenuating oxidative stress and inflammatory response in various disorders [23,24,25,26]. Nrf2 is a redox-sensitive transcription factor that regulates the transcription of a large number of cytodefensive genes, including heme oxygenase-1 (HO-1) and glutamate-cysteine ligase catalytic subunit (GCLC) [26]. Nrf2 is found sequestered in the cytoplasm by Kelch like ECH associated protein 1 (Keap1) and upon exposure to ROS or electrophiles, it dissociates and translocates to the nucleus where it binds the antioxidant response element (ARE) and activates the transcription of its target genes. The importance of Nrf2 activation in mitigating the severity of ALI has been demonstrated in numerous studies (reviewed in [27]). In addition to Nrf2, peroxisome proliferator-activated receptor gamma (PPARγ) is a ligand-activated transcription factor that can confer protection against oxidative stress and inflammatory response in respiratory diseases [28]. The upregulation of PPARγ mitigated chemotherapy-induced oxidative and inflammatory injury in rodent models [25,29,30] and can inhibit NF-κB via direct and indirect mechanisms [31].

The renin-angiotensin system (RAS) is a physiological regulator of blood pressure. It is involved in cardiovascular, renal, and adrenal functions primarily through the effects of angiotensin (Ang)-II [32]. The RAS is involved in inflammatory disorders and a significant interest in mitigating inflammation by drugs modulating this system has recently emerged [33]. In this context, Ang II AT1R blockers (ARBs) showed protective effects against inflammation, apoptosis, and endoplasmic reticulum (ER) stress in different experimental models [34,35,36,37]. Candesartan (CAN) is a highly selective Ang II AT1R blocker that showed beneficial effects against oxidative injury and inflammation [34,35]. CAN attenuated inflammation by inhibiting NF-κB activation and protected against stroke-induced neuronal damage [38], hypertension in pregnancy [39], and acute myocardial infarction (AMI) [35]. Despite the reported pharmacological effects of CAN, its ability to mitigate CIS-induced lung injury has not been reported yet. This study aimed to investigate the protective effect of CAN against CIS-induced oxidative stress, inflammation, and lung injury in rats, pointing to the involvement of TLR4/NF-κB, JAK1/STAT3, PPARγ, and Nrf2/HO-1 signaling.

## 2. Results

### 2.1. CAN Prevents CIS-Induced Lung Injury in Rats

**Figure 1:**
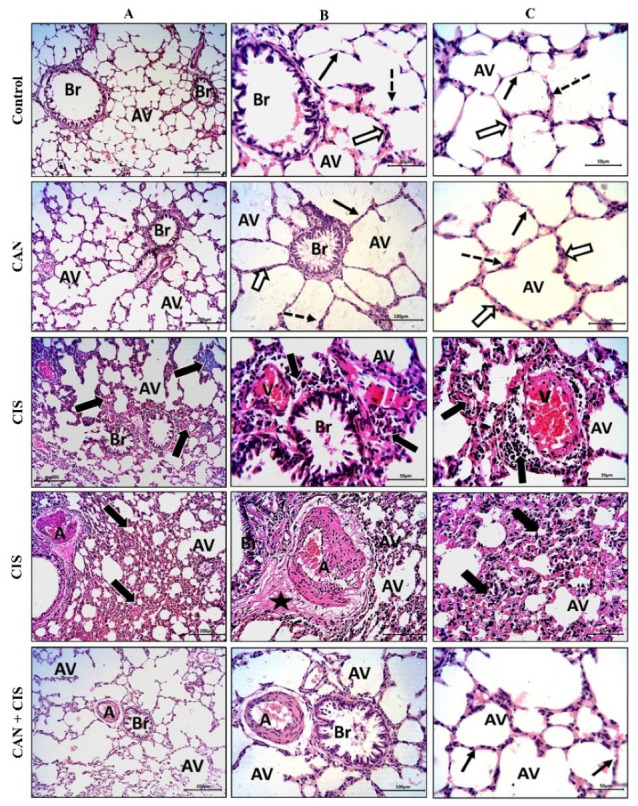
CAN prevented CIS-induced lung tissue injury. Photomicrographs of H&E-stained sections showing alveoli (AV) and bronchioles (Br) at ×100 (**A**), and at ×200 magnification (**B**,**C**). Control and CAN-treated rats showed normal alveoli lined with a thin type I alveolar epithelium (black arrows) with occasionally prominent rounded nuclei of type II cells (dotted arrows), thin interalveolar septa with no inflammatory cells (white arrows), and bronchioles (Br) with intact epithelium and lumen free of cell debris or secretions (Figure 1A–C). CIS-administered rats exhibited a decrease in the number of potent alveoli (Av), thick interalveolar septa with inflammatory cells (thick black arrows), congested veins (V) with perivascular mononuclear infiltrate (thick black arrows), thickened and deformed arteries (A), and perivascular edema (star). CAN-treated CIS-administered rats showed a significant improvement in the alveoli (AV), bronchioles (Br), and interalveolar septa (thin black arrows).

The histopathological examination of the alveoli and bronchioles of control and CAN-treated rats revealed normal alveoli lined with a thin type I alveolar epithelium with occasionally prominent rounded nuclei of type II cells, thin interalveolar septa with no inflammatory cells, and bronchioles with intact epithelium and lumen free of cell debris or secretions (Figure 1A–C). CIS decreased the number of potent alveoli and the interalveolar septa appeared thick with inflammatory cells. Congested veins with perivascular mononuclear infiltrate, thickened deformed arteries, and perivascular edema were observed in the lungs of CIS-treated rats (Figure 1A–C). CAN conferred a marked protection and prevented CIS-induced alveolar and bronchiolar injury (Figure 1A–C).

### 2.2. CAN Attenuates CIS-Induced Oxidative Stress in the Lungs of Rats

**Figure 2:**
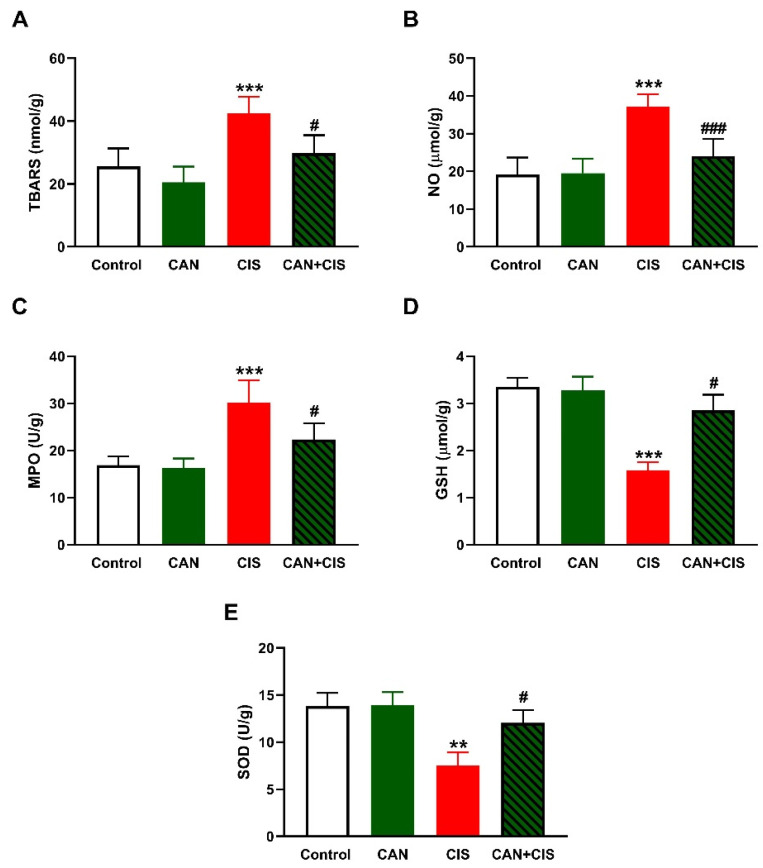
CAN attenuated CIS-induced oxidative stress in the lungs of rats. CAN decreased (**A**) TBARS, (**B**) NO, and (**C**) MPO activity, and increased (**D**) GSH and (**E**) SOD activity in CIS-administered rats. Data are Mean ± SEM, (*n* = 8). ** *p* < 0.01 and *** *p* < 0.001 vs. Control. # *p* < 0.05 and ### *p* < 0.001 vs. CIS.

LPO, assessed as thiobarbituric acid reactive substances (TBARS; Figure 2A), and nitric oxide (NO) (Figure 2B) levels, myeloperoxidase (MPO) activity (Figure 2C), GSH (Figure 2D), and SOD (Figure 2E) were determined to assess the effect of CAN on oxidative stress induced by CIS. TBARS, NO, and MPO were significantly increased (*p* < 0.001), whereas GSH (*p* < 0.001) and SOD (*p* < 0.01) were decreased in CIS-administered rats, as compared to the control. CAN decreased TBARS, NO, and MPO, and increased antioxidants in the lungs of CIS-treated rats whereas exerted no effect in normal rats.

### 2.3. CAN Suppresses TLR-4/NF-κB Signaling and Prevents Inflammation and Apoptosis in CIS-Administered Rats

**Figure 3:**
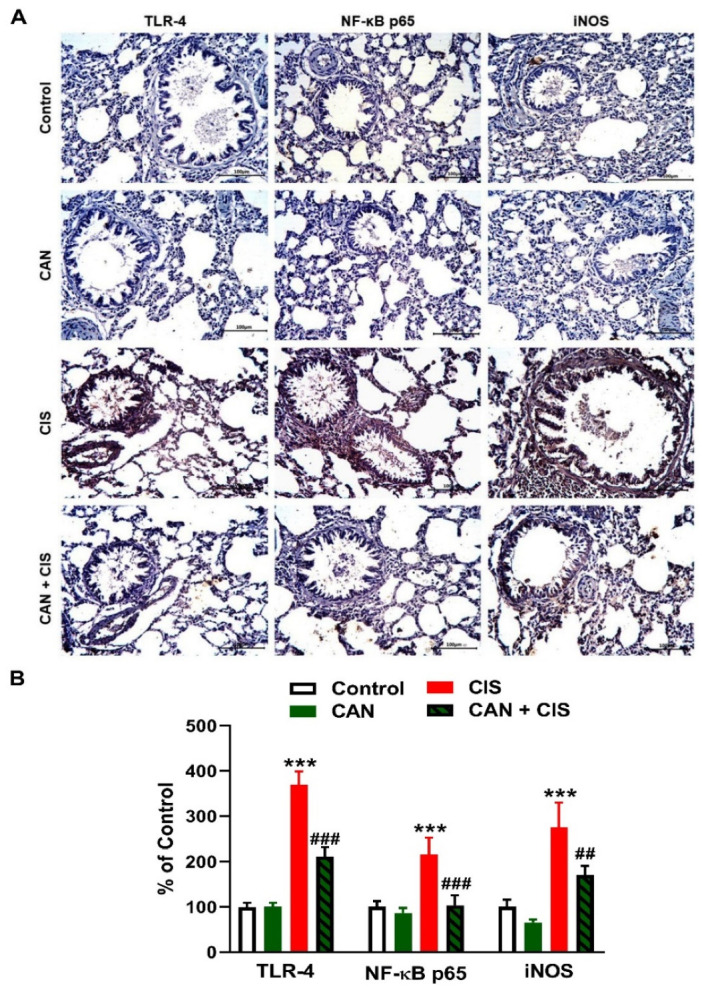
CAN downregulated TLR-4/NF-κB signaling in CIS-administered rats. (**A**) Photomicrographs of sections in the lungs of rats showing increased expression of TLR-4, NF-κB p65, and iNOS in CIS-administered rats and a marked decrease in CAN-treated rats. (**B**) Image analysis showed that CAN significantly decreased TLR-4, NF-κB p65, and iNOS in CIS-administered rats. Data are Mean ± SEM, (*n* = 8). *** *p* < 0.001 vs. Control, and ## *p* < 0.01 and ### *p* < 0.001 vs. CIS.

**Figure 4:**
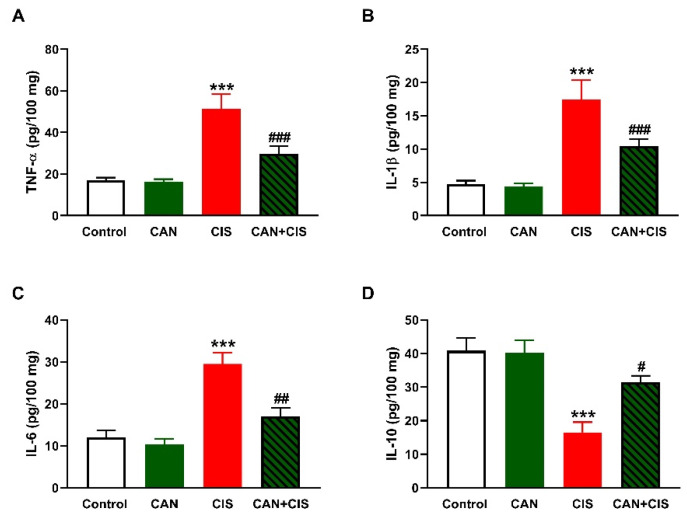
CAN decreased TNFα (**A**), IL-1β (**B**), and IL-6 (**C**), and increased IL-10 (**D**) in the lungs of CIS-administered rats. Data are Mean ± SEM, (*n* = 8). *** *p* < 0.001 vs. Control, and # *p* < 0.05, ## *p* < 0.01 and ### *p* < 0.001 vs. CIS.

**Figure 5:**
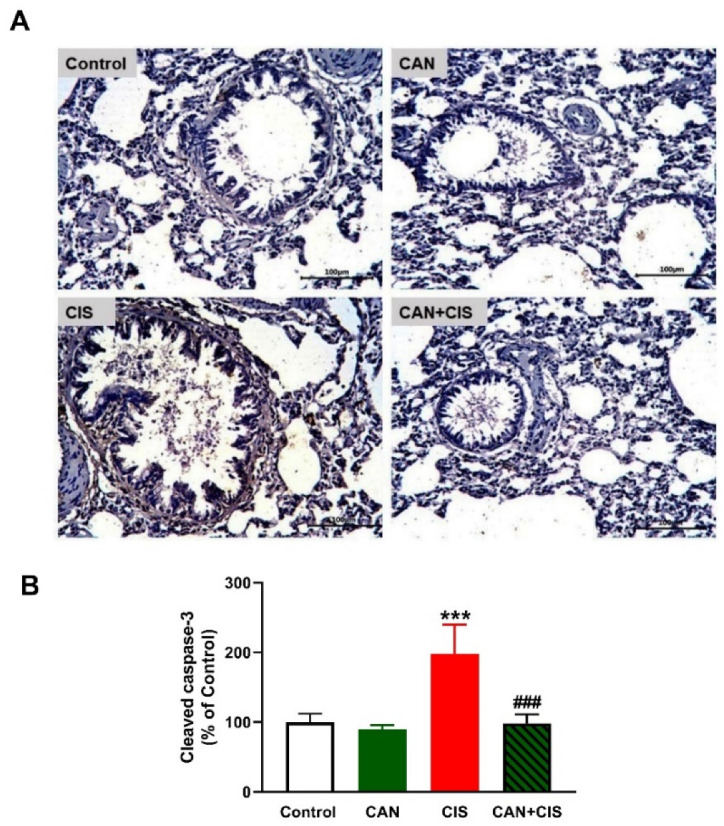
CAN downregulated cleaved caspase-3 in CIS-administered rats. (**A**) Photomicrographs of sections in the lungs of rats showing increased expression of cleaved caspase-3 in CIS-administered rats and a marked decrease in CAN-treated rats. (**B**) Image analysis showed that CAN significantly decreased cleaved caspase-3 in CIS-administered rats. Data are Mean ± SEM, (*n* = 8). *** *p* < 0.001 vs. Control and ### *p* < 0.001 vs. CIS.

Changes in the expression of TLR-4, NF-κB p65, iNOS, and cytokines were determined to evaluate CIS-induced inflammation and the protective effect of CAN. Immunohistochemical (IHC) analysis revealed a significant upregulation of TLR-4, NF-κB p65, and iNOS in the lungs of rats that received CIS (Figure 3A,B), as compared to the control (*p* < 0.001). CAN effectively downregulated tTLR-4 (*p* < 0.001), NF-κB p65 (*p* < 0.001), and iNOS (*p* < 0.01) in the lungs of CIS-treated rats while had no effect in normal rats. Likewise, the pro-inflammatory cytokines TNF-α (Figure 4A), IL-1β (Figure 4B), and IL-6 (Figure 4C) were elevated (*p* < 0.001) and the anti-inflammatory IL-10 was decreased (Figure 4D; *p* < 0.001) following CIS administration. Treatment with CAN ameliorated the levels of cytokines in the lungs of CIS-administered rats. CIS has also increased the expression of cleaved caspase-3 in the lungs of rats (Figure 5A,B), as compared to the control (*p* < 0.001), an effect that was reversed in CAN-treated rats.

### 2.4. CAN Ameliorates Ang II and Ang (1–7) in CIS-Administered Rats

**Figure 6:**
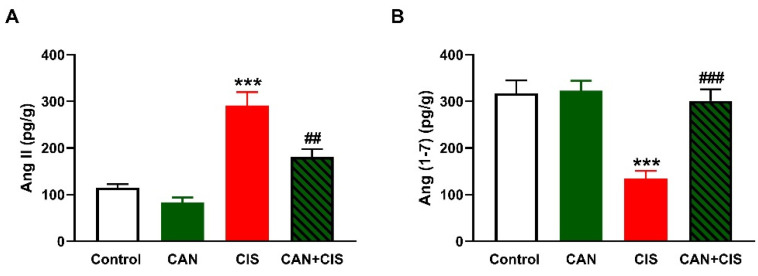
CAN decreased Ang II (**A**) and increased Ang (1–7) (**B**) in the lungs of CIS-administered rats. Data are Mean ± SEM, (*n* = 8). *** *p* < 0.001 vs. Control, and ## *p* < 0.01 and ### *p* < 0.001 vs. CIS.

As depicted in Figure 6A,B, CIS increased Ang II and decreased Ang (1–7), respectively, in the lungs of rats (*p* < 0.001). While CAN exerted a non-significant effect in normal rats, it decreased Ang II (*p* < 0.01) and increased Ang (1–7) (*p* < 0.001) in CIS-intoxicated rats.

### 2.5. CAN Downregulates JAK1/STAT3 Signaling in CIS-Administered Rats

**Figure 7:**
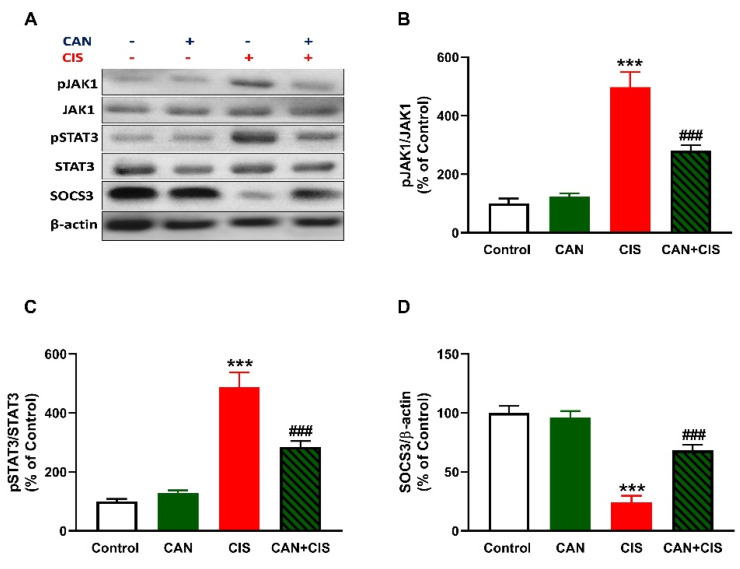
CAN downregulated JAK1/STAT3 signaling in CIS-administered rats. (**A**) Representative blots of phosphorylated and total JAK1 and STAT3, SOCS3, and β-actin. (**B**–**D**) CAN decreased JAK1 phosphorylation (**B**) and STAT3 phosphorylation (**C**), increased SOCS3 (**D**) in the lungs of CIS-administered rats. Data are Mean ± SEM, (*n* = 8). *** *p* < 0.001 vs. Control, and ### *p* < 0.001 vs. CIS.

CIS administration increased JAK1 and STAT3 phosphorylation in the lungs of rats (*p* < 0.001), as depicted in Figure 7A–C. In contrast, SOCS3 was significantly downregulated in the lungs of rats that received CIS (Figure 7A,D; *p* < 0.001). CAN markedly decreased JAK1 and STAT3 phosphorylation and increased SOCS3 in CIS-administered rats (*p* < 0.001). Of note, CAN did not affect JAK1/STAT3 signaling in normal rats (Figure 7).

### 2.6. CAN Upregulates Nrf2/HO-1 Signaling and PPARγ in CIS-Administered Rats

**Figure 8:**
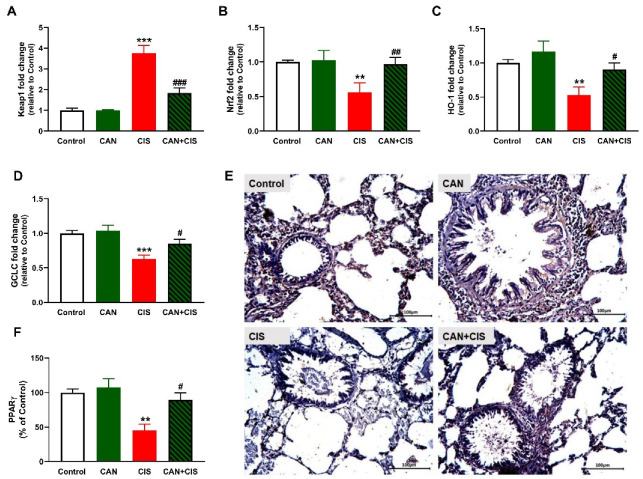
CAN upregulated Nrf2/HO-1 signaling and PPARγ in CIS-administered rats. CIS decreased Keap1 (**A**), and increased Nrf2 (**B**), HO-1 (**C**), and GCLC (**D**) mRNA abundance in the lungs of CIS-administered rats. Photomicrographs of the lung sections (**E**) and image analysis (**F**) showed an increase in PPARγ expression in CIS-administered rats treated with CAN. ** *p* < 0.01 and *** *p* < 0.001 vs. Control. # *p* < 0.05, ## *p* < 0.01 and ### *p* < 0.001 vs. CIS.

The determination of mRNA abundance of Keap1 revealed a significant increase in the lungs of CIS-administered rats (Figure 8A), as compared to the control (*p* < 0.001). Nrf2 (Figure 8B), HO-1 (Figure 8C) and GCLC (Figure 8D) were declined significantly in CIS-administered rats. CAN markedly decreased Keap1 and upregulated Nrf2, HO-1, and GCLC in the lung of CIS-administered but not in normal rats. IHC showed a significant decrease in PPARγ in rats that received CIS (*p* < 0.01; Figure 8E,F), an effect that was notably reversed in CIS-treated rats (*p* < 0.05).

## 3. Discussion

Cisplatin (CIS) is one of the most effective chemotherapeutics but adverse effects, including ALI, confine its clinical applications [3,4]. Oxidative stress and injurious inflammatory responses have been implicated in CIS toxicity [7,40]. The Ang II AT1R blocker CAN showed beneficial effects against inflammation and stress in cardiovascular, neurovascular, and other diseases [34,35,38,39]. Herein, we investigated the protective effect of CAN against oxidative stress, inflammation, and lung injury induced by CIS in rats, emphasizing the role of TLR4/NF-κB, JAK1/STAT3, PPARγ, and Nrf2/HO-1 signaling.

CIS caused lung injury manifested by the histopathological changes in the bronchi and lungs, including inflammatory changes, inflammatory exudates, thickening of the interalveolar septa, venous congestion, deformed and thickened arterial wall, and perivascular edema. CIS-induced histopathological alterations, such as alveolar septal fibrosis, leukocyte infiltration, hemorrhage, alveolar edema, and severe alveolar damage have been previously observed, pinpointing its pulmonary toxicity [8,9]. These changes were potently ameliorated by the administration of CAN, establishing its protective effect against CIS pulmonary intoxication.

Owing to the role ROS and inflammation play in CIS-induced lung injury [8,9,10,11], we assumed that amelioration of oxidative stress and inflammation mediated, at least in part, the protective effect of CAN. In support of previous studies [8,9], CIS caused oxidative stress evidenced by elevated TBARS, NO, and LPO along with decreased GSH and SOD. CIS increases ROS production which can provoke damage to cellular macromolecules, including peroxidation of the membrane lipids, leading to disrupted membrane fluidity and permeability. In addition, ROS can deplete GSH and antioxidant enzymes via their versatile oxidative activity [41]. NO can react with superoxide and the produced peroxynitrite is a very potent oxidant that can damage DNA and promote further production of ROS [42]. MPO is produced by neutrophils and catalyzes the formation of reactive oxidant species that are capable of eliciting both LPO and nitration, oxidative cross-linking, and halogenation of proteins [43]. MPO is thought to be directly implicated in lung injury and inflammation and is often increased in many lung pathologies [44]. In addition to their induced deleterious effects on cellular macromolecules, ROS activate TLR-4 and subsequently NF-κB and the release of inflammatory mediators, including iNOS, TNF-α, IL-1β, and IL-6 [12,13,14]. In this study, CIS upregulated TLR-4, NF-κB, iNOS, and pro-inflammatory cytokines, and decreased the anti-inflammatory cytokine IL-10. These findings demonstrated the development of an inflammatory response in the lungs of CIS-intoxicated rats. The role of TLR-4 in promoting inflammation in ALI has been well-acknowledged [15,16]. The upregulation of iNOS explained the increase in NO levels following CIS administration. The released cytokines along with ROS can provoke apoptosis via eliciting the loss of mitochondrial membrane potential and the release of cytochrome c [45]. In the cytosol, cytochrome c promotes a series of processes leading to the activation of caspase-3 that elicit the degradation of DNA and cellular and cytoskeletal proteins, culminating in cell death [46]. Our findings showed a remarkable activation of caspase-3 in the lungs of CIS-intoxicated rats.

CAN effectively suppressed LPO, NO, and MPO activity and enhanced GSH and SOD in CIS-administered rats. In addition, CAN downregulated TLR-4, NF-κB p65, iNOS, pro-inflammatory cytokines, and caspase-3 while IL-10 was upregulated in the lungs of CIS-treated rats. These findings provided evidence of the protective effect of CAN against CIS-induced oxidative stress and inflammation which has been shown in previous studies. CAN effectively inhibited NADPH oxidase and the production of ROS in fatty acid-treated pancreatic β-cells [47], and protected the kidney against hyperglycemia-associated oxidative stress in diabetic rats [48]. In the experimental models of AMI [35], stroke-induced neuronal damage [38], and hypertension in pregnancy [39], CAN inhibited NF-κB and suppressed inflammation. Inhibition of TLR-4 signaling has been demonstrated as a mechanism mediating the anti-inflammatory activity of CAN. In hypertensive rats, CAN as well as the TLR-4 inhibitor VIPER downregulated TLR-4, MyD88, TNF-α, IL-1β, and NF-κB, and increased the anti-inflammatory cytokine IL-10 in the rostral ventrolateral medulla [49]. CAN modulated microglia activation and polarization [38], and protected human monocytes and mice against LPS-induced inflammation [50] by inhibiting TLR-4/NF-κB signaling. Our study added support to these findings by showing the ability of CAN to modulate TLR-4/NF-κB signaling and prevent CIS-induced lung injury in rats.

Given the involvement of RAS in promoting oxidative stress and inflammation [33,51], it is noteworthy assuming that CAN attenuated inflammation by blocking Ang II AT1R receptors. In addition to its vasoconstrictive effect, Ang II acts as a pro-inflammatory component by eliciting the release of inflammatory mediators from the endothelium [52] and smooth muscle cells [53]. In renal tubular cells of rats. Ang II upregulated TLR-4 signaling and the cytokines TNF-α and IL-1β [54]. Ang II was significantly elevated in CIS-administered rats in this study, whereas Ang 1–7 was decreased. Ang 1–7 counteracts the Ang II/AT1R axis and induces anti-inflammatory, vasodilating, anti-angiogenic, and antihypertensive activities [55]. Our study introduced new information that CAN decreased Ang II and boosted Ang (1–7) in the lungs of CIS-treated rats, findings supporting the studies showing its ability to modulate ACE2-Ang (1–7)-mas axis in the heart of murine models of cardiotoxicity [36] and pressure overload-induced cardiac remodeling [37].

In addition to TLR-4/NF-κB signaling and Ang II/AT1R axis, CAN downregulated JAK1/STAT3 signaling in the lungs of CIS-administered rats. CIS upregulated JAK1 and STAT3 phosphorylation and downregulated SOCS3 in the lungs of rats, an effect that could be attributed to oxidative stress and cytokines, in particular IL-6 [19,20,21,22]. IL-6 mediates different inflammatory responses and STAT3 was activated in the fibroblasts isolated from normal and fibrotic lungs after its binding to the receptor gp130 [56,57]. In a mouse model of bleomycin-induced pulmonary fibrosis, JAK1 has been overexpressed and its active form was increased in the lungs [58]. The JAK/STAT pathway plays a critical role in inflammation and its abnormal activation causes an excessive inflammatory response [17,18]. JAK can activate NF-κB by affecting the activity of other kinases such as Raf-1, MAPK and inhibiting I-κB phosphorylation and degradation [59]. The phosphorylated STAT3 promotes the recruitment of inflammatory cells thereby amplifying the inflammatory response [17,18]. Therefore, the suppression of JAK1 and STAT3 phosphorylation and upregulation of SOCS3 were involved in the anti-inflammatory activity of CAN. SOCS3 regulates the phosphorylation of JAK by targeting the IL-6 family cytokine-receptor complex, including gp130. The SH2 domain of SOCS3 binds to gp130 p-Tyr759 followed by binding of the Ig-like receptors to gp130-related JAK and hiding the substrate-binding groove of JAK, resulting in JAK/STAT signaling inhibition [60].

To further explore the mechanism(s) underlying the protective effect of CAN against CIS-induced oxidative and inflammatory responses in the lungs, we evaluated the changes in Nrf2/HO-1 signaling and PPARγ. CIS injection upregulated Keap1, and downregulated Nrf2, HO-1, GCLC, and PPARγ in the lungs of rats, effects that were reversed in the CAN-treated rats. CAN activated Nrf2 and its regulated genes and decreased its suppressor Keap1. The activation of Nrf2 in the lungs of CIS-administered rats treated with CAN explained the improved antioxidant defenses and the attenuation of oxidative stress and inflammation. Nrf2 and HO-1 can directly inhibit NF-κB and the inflammatory response along with activating the anti-inflammatory mechanisms, thereby regulating the inflammatory cascade [61]. Previous studies have demonstrated the beneficial effects of Nrf2 activation in attenuating chemotherapy-induced toxicity, including ALI [23,24,25,27]. Nrf2 activation has also inhibited TLR-4 and the inflammatory cascade in a murine model of ischemia-reperfusion (I/R)-induced lung injury [62]. In addition to Nrf2, CAN upregulated PPARγ in CIS-intoxicated rats. PPARγ activation protected the lungs and other organs [25,29,30]. PPARγ upregulates the expression of antioxidant genes [63] and inhibits NF-κB via direct and indirect mechanisms [31]. The anti-inflammatory effect of PPARγ has been mediated via inhibiting TLR-4 and NF-κB in LPS-stimulated macrophages [64] and tobacco smoke-induced alveolar macrophages [65]. The attenuation of oxidative stress and inflammation via CAN-induced activation of Nrf2 and PPARγ in the lungs of CIS-administered rats might involve the suppression of JAK/STAT signaling. This notion is supported by studies showing that the attenuation of ROS production was associated with suppression of JAK/STAT in vivo [20,21,22], and that PPARγ suppressed the JAK/STAT-dependent inflammatory responses by upregulating SOCS3 in glial cells [66].

## 4. Materials and Methods

### 4.1. Drugs and Chemicals

CAN was purchased from AstraZeneca (Cairo, Egypt). The ELISA kits for rat TNF-α, IL-6, IL-β, IL-10, Ang-II, and Ang-(1–7), and antibodies for SOCS3 (Cat. no. E-AB-63265), p-JAK-1 (Cat. no. E-AB-20913), and β-actin (Cat. no. E-AB-20031) were purchased from ELabscience (Wuhan, China). The antibodies for NF-κB p65 (Cat. no. BBP1066), TLR-4 (Cat. no. YPA2203), iNOS (Cat. no. YPA1072), PPAR-γ (Cat. no. YPA2204), cleaved-caspase-3 (Cat. no. YPA2210) were supplied by Biospes (Chongqing, China), and JAK1 (Cat. no. sc-136225), STAT3 (Cat. no. sc-293151), and p-STAT3 (Cat. no. sc-81523) were obtained from Santa Cruz Biotechnology (Dallas, TX, USA).

### 4.2. Experimental Animals and Treatments

Twelve-week-old male Wistar rats (180–210 g) were obtained from the central animal house (Faculty of Medicine, Assiut University) and housed in clean plastic cages under optimal laboratory conditions (temperature of 23 ± 1 °C, relative humidity of 50–60%, and a 12 h dark/light cycle).

Thirty-two rats were randomly allocated into four groups (*n* = 8):

Group I (Control): were given vehicle (saline) orally for 10 days and a single intraperitoneal (i.p.) injection of saline at day 6.

Group II (CAN): were given 5 mg/kg CAN [67] dissolved in saline orally for 10 days and a single i.p. injection of saline at day 6.

Group III (CIS): were given saline orally for 10 days and a single i.p. injection of 7 mg/kg CIS dissolved in saline [6] at day 6.

Group IV (CAN + CIS): were given 5 mg/kg CAN orally for 10 days and a single i.p. injection of CIS (7 mg/kg) dissolved in saline at day 6.

Twenty-four hours after the last treatment, the rats were anesthetized with ketamine (100 mg/kg), sacrificed, dissected and the lungs were removed and washed in cold saline. Samples from the lungs were fixed in 10% neutral-buffered formalin (NBF). Other samples were stored at −80 °C, and others were homogenized (10% *w*/*v*) in Tris-HCl buffer (pH = 7.4). The homogenate was centrifuged, and the supernatant was stored at −80 °C.

### 4.3. Histological and Immunohistochemical (IHC) Assessment

The lung specimens were fixed in 10% NBF for 24 h, washed, dehydrated, cleared, and embedded in paraffin. 4-µm sections were cut using a rotary microtome, mounted on glass slides, and processed for hematoxylin and eosin (H&E) staining [68]. The slides were examined with an Olympus^®^ light microscope. Other sections were deparaffinized and antigen retrieval with carried out using 0.05 M citrate buffer (pH = 6.8) followed by blocking with 1% bovine serum albumin (BSA) and treatment with 0.3% hydrogen peroxide (H_2_O_2_). Following the washing in phosphate-buffered saline PBS, the slides were incubated with primary antibodies for NF-κB p65, iNOS, PPAR-γ, and cleaved-caspase-3 overnight at 4 °C. The slides were washed and incubated with the secondary antibodies for 1 h at room temperature and the color was developed through incubation with DAB in H_2_O_2_. Hematoxylin was used for counter-staining and the slides were examined under a light microscope. The images were captured, and the color intensity was determined using ImageJ (NIH, Bethesda, MD, USA).

### 4.4. Biochemical Assays

TBARS, GSH, and NO were assayed in the lung homogenate following the methods described by Mihara and Uchiyama [69], Ellman [70], and Montgomery and Dymock [71], respectively. SOD and MPO activities were determined according to Marklund and Marklund [72] and Krawisz et al. [73], Respectively. TNF-α, IL-1β, IL-6, IL-10, Ang-II, and Ang-(1–7) levels were determined in the lung homogenate using specific ELISA kits, according to the manufacturer’s instructions.

### 4.5. q-RT-PCR Analysis

The quantity and quality of RNA were determined using a spectrophotometer after the extraction of total RNA from the lung tissues using Trizol (ThermoFisher, Waltham, MA, USA). The RNA samples with OD260/OD280 ≥ 1.8 were reverse transcribed into cDNA using a reverse transcription kit (ThermoFisher, Waltham, MA, USA). The cDNA was amplified using SYBR green Master Mix (ThermoFisher, USA) and the primer pairs (Vivantis Technologies, Selangor, Malaysia) listed in Table 1. The obtained data were analyzed using 2^−∆∆Ct^ method [74] and normalized to GAPDH.

### 4.6. Western Blotting

The frozen lung samples were homogenized in RIPA buffer with a phosphatase/proteinase inhibitor cocktail. The homogenate was centrifuged, and protein content was determined in the supernatant using Bradford reagent. Thirty µg protein was subjected to SDS-PAGE and the separated protein bands were transferred to PVDF membranes. Following the blocking with 5% BSA, the membranes were incubated with primary antibodies against JAK1, p-JAK-1, STAT3, p-STAT3, SOCS3, and β-actin overnight at 4 °C. The membranes were washed and incubated at 4 °C with the appropriate secondary antibody for 1 h at RT. After washing, the bands were developed using a BCIP/NBT substrate detection kit (GeneMed Biotechnologies, San Francisco, CA, USA) and the band intensity was determined using ImageJ.

### 4.7. Statistical Analysis

The data were presented as means ± standard error of the mean (SEM). One-way ANOVA followed by a Tukey’s test were used to determine statistical significance among the experimental groups on GraphPad 8. A p value less than 0.05 was considered statistically significant

## 5. Conclusions

The findings of this investigation provide new information about the protective effect of CAN against CIS-induced lung injury and the possible underlying mechanism. CAN prevented lung tissue injury, oxidative stress, inflammatory responses, and apoptosis, and enhanced the antioxidant defenses in CIS-administered rats. The beneficial effect of CAN was associated with the suppression of TLR-4/NF-κB and JAK1/STAT3 signaling pathways and Ang II/AT1R axis. In addition, CAN upregulated PPARγ and Nrf2/HO-1 signaling in the lungs of CIS-administered rats. Therefore, CAN can confer protection against CIS-induced ALI, giving a preference of using CIS as an effective anti-neoplastic agent with minimal fear of pulmonary injury.

## Figures and Tables

**Table 1:** Primers used for qRT-PCR.

Gene	Forward Primer (5′-3′)	Reverse Primer (5′-3′)
Nrf2	TTGTAGATGACCATGAGTCGC	TGTCCTGCTGTATGCTGCTT
GCLC	GTTGTTACTGAATGGCGGCG	CGGCGTTTCCTCATGTTGTC
HO-1	GTAAATGCAGTGTTGGCCCC	ATGTGCCAGGCATCTCCTTC
Keap1	TCAGCTAGAGGCGTACTGGA	TTCGGTTACCATCCTGCGAG
GAPDH	TGCTGGTGCTGAGTATGTCG	TTGAGAGCAATGCCAGCC

## Data Availability

The data presented in this study are available in article.
